# Poxvirus cGAMP nucleases: Clues and mysteries from a stolen gene

**DOI:** 10.1371/journal.ppat.1009372

**Published:** 2021-03-18

**Authors:** Carlos Maluquer de Motes

**Affiliations:** Department of Microbial Sciences, School of Biosciences and Medicine, University of Surrey, Guildford, United Kingdom; Mount Sinai School of Medicine, UNITED STATES

## Introduction

**Fig 1:**
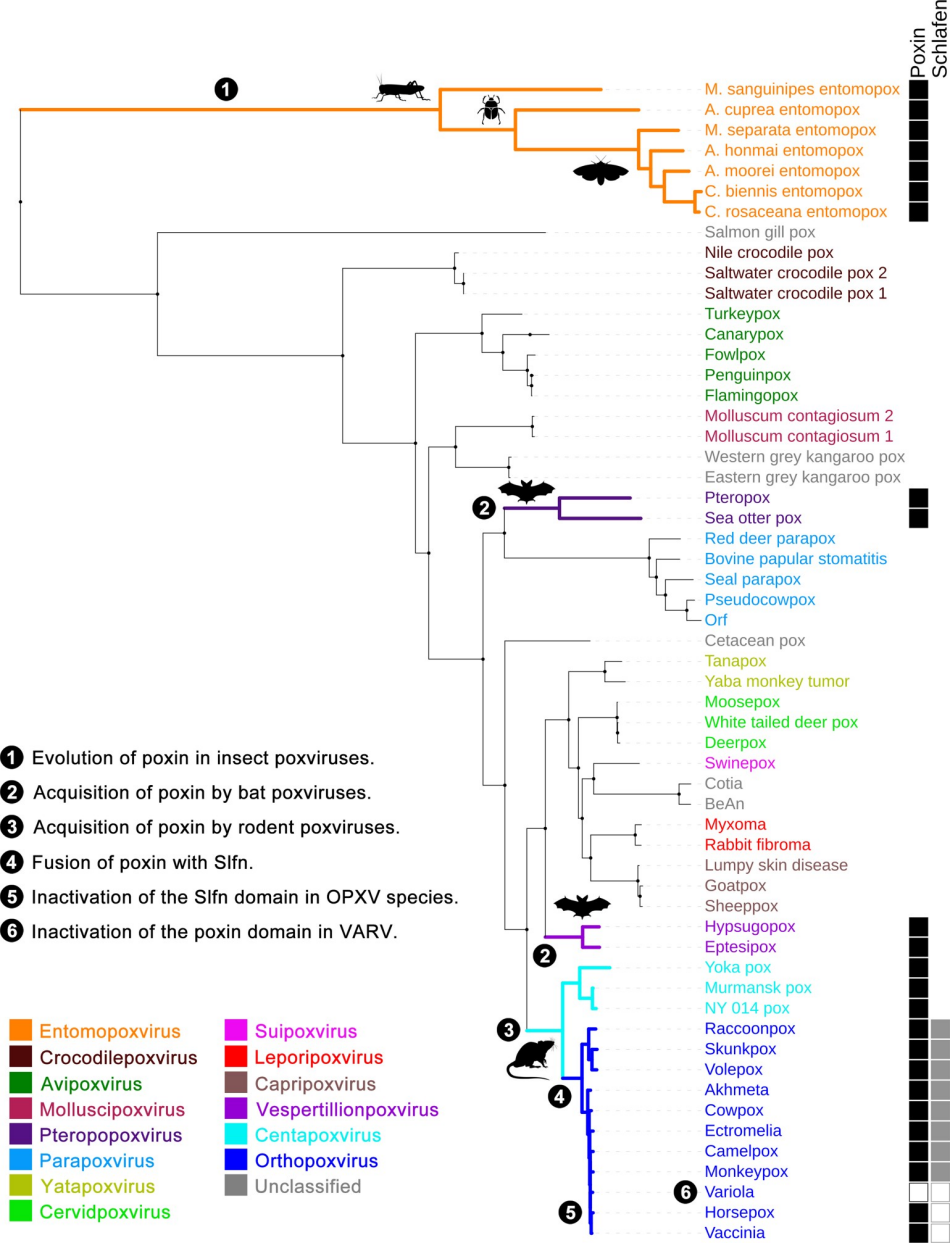
Distribution of cGAMP nucleases across poxvirus species and genera. A representative maximum-likelihood phylogenetic tree for the Poxviridae family was generated from a multiple sequence alignment of the DNA-dependent RNA polymerase subunit 147 kDa protein (RPO147, *J6R*) from reference species, colour-coded according to their current classification. Accession numbers were as follows: vaccinia virus, AY243312; horsepox virus, DQ792504; variola virus, X69198; monkeypox virus, AY603973; camelpox virus, AF438165; ectromelia virus, NC_004105; cowpox virus, NC_003663; akhmeta virus, MH607143; volepox virus, KU749311; skunkpox virus, KU749310; raccoonpox virus, KP143769; NY poxvirus, MF001305; Murmansk poxvirus, MF001304; Yoka poxvirus, HQ849551; eptesipox virus, NC_035460; hypsugopox virus, MK860688; sheeppox virus, NC_004002; goatpox virus, AY077835; lumpy skin disease virus, AF409137; rabbit fibroma virus, AF170722; myxoma virus, AF170726; BeAn virus, KY094066; Cotia virus, KM595078; swinepox virus, AF410153; deerpox virus, AY689436; white tailed deer poxvirus, MF966153; moosepox virus, MG751778; Yaba monkey tumor virus, AY386371; tanapox virus, EF420156; cetacean poxvirus, MN653921; orf virus, AY386264; pseudocowpox virus, NC_013804; seal parapox virus, KY382358; bovine papular stomatitis virus, AY386265; red deer parapox virus, KM502564; sea otter poxvirus, MH427217; pteropox virus, KU980965; eastern grey kangaroo poxvirus, MF661791; western grey kangaroo poxvirus, MF467280; molluscum contagiosum virus 1, NC_001731; molluscum contagiosum virus 2, MH320549; flamingopox virus, MF678796; penguinpox virus, KJ859677; fowlpox virus, AF198100; canarypox virus, AY318871; turkeypox virus, KP728110; saltwater crocodile poxvirus 1, MG450915; saltwater crocodile poxvirus 2, MG450916; Nile crocodile poxvirus, DQ356948; salmon gill poxvirus, KT159937; *C*. *rosaceana* entomopox virus, HF679133; *C*. *biennis* entomopox virus, HF679132; *A*. *moorei* entomopox virus, AF250284; *A*. *honmai* entomopox virus, HF679131; *M*. *separata* entomopox virus, HF679134; *A*. *cuprea* entonomopox virus, AP013055; *M*. *sanguinipes* entomopox virus, NC_001993. Sequences were manually retrieved and aligned using Molecular Evolutionary Genetics Analysis (MEGA) package. This multiple sequence alignment was used to construct the phylogenetic tree. The presence of poxin in a lineage is indicated by coloured branches and a solid black box on the right-hand side. The presence of a Slfn domain is indicated by a solid grey box next to the poxin box. Empty boxes indicate gene inactivation (defined by the presence of at least one premature STOP codon truncating the predicted open reading frame).

Over the last few years, our understanding of the role of cyclic GMP-AMP (cGAMP) has rapidly expanded, and this second messenger is now acknowledged to be a major driver of antiviral responses in animals as well as in bacteria [1–3]. While in bacteria cGAMP belongs to an expanding family of cyclic nucleotides, metazoans seem to be restricted to the production of cGAMP in its 2′3′ form (containing G(2′,5′)pA and A(3′,5′)pG phosphodiester linkages) by the DNA-binding enzyme cGAMP synthase (cGAS). cGAMP binds and activates the signalling adaptor Stimulator of Interferon Genes (STING), rapidly amplifying immune pathways resulting in interferon (IFN) production and thereby holding a prominent role in the mammalian antiviral response. Unlike most DNA viruses that replicate inside the cell nucleus avoiding the hostile cytosolic environment, poxviruses, a large family of linear dsDNA viruses, complete their life cycle exclusively in the cytoplasm. Such a strategy requires poxviruses to encode effective immune evasion mechanisms that dampen cellular cytosolic DNA surveillance, and a number of these have been described [4,5]. Recently, the immunomodulatory capacity of poxviruses gained a new perspective with the discovery of a viral cGAMP nuclease termed poxin (poxvirus immune nuclease) [6]. Poxins are the first type of cytosolic proteins able to cleave the 3′-5′ bond of cGAMP, generating a linear version unrecognised by STING. The immunological impact of cGAMP during poxvirus infection is enormous. In ectromelia virus (ECTV), the causative agent of mousepox, the absence of poxin activity results in a complete inability to prevent STING activation in cells and a 5-log attenuation of the virus in mice [7]. Such a dramatic attenuation after single deletion of an immunomodulatory gene is remarkable and demonstrates the critical role of cGAMP in the immune response against poxvirus infection. Somewhat surprising therefore is that poxin homologues are not conserved across the different poxvirus genera and they appear in different forms. While poxin is widely found in insect poxviruses, it is missing in bird, reptile, and fish poxviruses (which cluster at the base of the group and indicate that poxin was not present in ancestral poxviruses) and is only found in bat and rodent poxviruses, which subsequently acquired it by horizontal transfer (Fig 1). Poxvirus cGAMP nucleases have thus followed a unique evolutionary history that reflects their host and ecological niche and informs us of the importance of DNA sensing and cGAMP responses in those hosts.

### Acquisition of cGAMP nucleases by insect poxviruses

Poxin was originally identified as the product of the vaccinia virus (VACV) gene *B2R*. Bioinformatic searches surprisingly retrieved poxin homologues in entomopoxviruses and baculoviruses, where the gene is commonly known as p26, as well as their lepidopteran hosts such as butterflies and moths [6]. The presence of poxin homologues in insects and insect viruses, rather than mammals and mammalian viruses, suggested an invertebrate origin for cGAMP nucleases. Recent work has revealed a widespread distribution of poxin-like enzymes with capacity to degrade 2′3′-cGAMP in invertebrate DNA and RNA viruses and their hosts [8]. These remarkable findings uncover a vast source of cGAMP nucleases that permitted successful horizontal transfer to insect poxviruses and reveal the importance of cGAMP in insects. Structural comparisons revealed close homology between cGAMP nucleases and (+)ssRNA viral proteases. Some of these proteases were also able to cleave cGAMP [8], suggesting that this ability was evolutionarily exploited when these genes were acquired by insect DNA viruses and their hosts where they diverged originating the poxin gene family. Recently, cGAMP has been shown to drive a potent antiviral response via STING and the NF-κB transcription factor Relish in *Drosophila* [9]. An important question that arises from this work is what enzyme(s) are producing cGAMP in *Drosophila*, dipterans, and insects? Although insects encode nucleotide cyclases, it is unclear whether these can synthesise cGAMP. It is also unclear whether these enzymes will bind and become activated by cytosolic DNA given that insect RNA viruses also encode cGAMP nucleases. The search for these noncanonical cGAMP producing enzymes will provide unique evolutionary insights into insect immunity and perhaps yield unexpected connections with cGAS-like enzymes in prokaryotes, where cytosolic DNA is unlikely to be the (only) trigger [1].

### cGAMP nucleases in bat poxviruses

Insect poxviruses infecting orthopterans, hymenopterans, coleopterans, dipterans, and lepidopterans have been identified. Lepidopteran poxviruses and their hosts are by far the largest group containing cGAMP nucleases. Lepidopterans are prey for bats, and the bat-lepidopteran arms race has escalated into remarkable evolutionary adaptations. Therefore, their insectivorous nature may have facilitated transfer of poxin from lepidopteran to bat poxviruses. All 3 bat poxvirus species for which the complete genome sequence exists (Eptesipox virus [EPTV], Hypsugopox virus [HYPV], and Pteropox virus [PTPV] [10–12]) contain a cGAMP nuclease gene, and EPTV and HYPV were isolated from insect-eating bats. PTPV was isolated from a frugivorous bat native to Australia and is not closely related to EPTV and HYPV (Fig 1), so the acquisition of poxin by PTPV remains more enigmatic and may represent an independent event. Bats have evolved conserved pattern-recognition receptors able to detect viral RNA and DNA [13]. Studies indicate that bats trigger robust IFN responses to RNA virus infection, but responses to DNA viruses are dampened. Dampened DNA sensing pathways may also reduce self-DNA recognition occurring upon DNA damage caused by their unique nature as flying mammals. Indeed, bat STING is mutated at position 358 leading to reduced IFN production [14]. While bat poxviruses need to antagonise RNA detection systems such as RIG-I-like receptors and protein kinase R, the acquisition and retention of cGAMP nucleases in bat poxviruses demonstrates that they also need to antagonise DNA detection systems and that, despite being weakened, antiviral DNA sensing in bats must be effective. In addition, unlike many bat viruses, these poxviruses are associated with disease, which suggests that cGAMP degradation may contribute to virulence and reveal the importance of cGAMP in the bat antiviral response. Elucidating the specific adaptations evolved by bats to retain effective sensing of DNA viruses while minimising self-DNA detection will reveal how bats resist or succumb to virus infection and the viral dynamics and disease associations that occur in wild bat populations.

### vSchlafen—A marker for rodent tropism?

In addition to bat poxviruses, poxin can be found in the centapoxvirus and orthopoxvirus (OPXV) genera, which are phylogenetically related and share a common ancestor (Fig 1). Although still obscure, the natural host(s) and reservoir of centapoxviruses like the OPXV are thought to be rodents. It is therefore likely that the common centapoxvirus and OPXV ancestor infected an insectivorous rodent and acquired poxin from insect poxviruses. Poxin evolved as a single gene in centapoxviruses, but not in the OPXV where it fused to a second gene with homology to the mammalian family of schlafen (Slfn) proteins, becoming a viral Schlafen (vSlfn) [6,7,15]. The immunological functions of the murine and human Slfn genes remain largely unknown, and our work with the ECTV vSlfn showed no contribution to virulence when the Slfn domain was expressed on its own [7]. vSlfn is, however, highly conserved among OPXV, and its fusion to poxin in the only DNA viruses that replicate exclusively in the cytoplasm strongly suggests a role in DNA sensing and/or immune evasion, thus warranting further investigation. OPXV include the obligate human pathogen variola virus (VARV), as well as the zoonotic pathogens: monkeypox virus (MPXV) and cowpox virus (CPXV). The poxin-Slfn fusion is conserved in most OPXV, particularly those like MPXV, CPXV, and ECTV originating from a rodent reservoir. The Slfn domain is, however, mutated in the OPXV that no longer require rodent transmission such as VACV or the now extinct horsepox virus or VARV. Inactivation of the Slfn domain may thus correlate with adaptation to other hosts, including humans. Fundamental differences between human and mouse cGAS and DNA sensing exist, and retention of the full vSlfn may reflect the need to counteract immune constraints affecting poxvirus transmission in rodents, but not in humans. The signature of encoding a complete vSlfn may thus be a marker for rodent species tropism, which could become a valuable tool to ascertain the capacity of human-to-human transmission of zoonotic OPXV like MPXV.

### Inactivation of poxin

Surprisingly, the 2 poxvirus species that contain an inactivated poxin gene are arguably the most attenuated and the most virulent. Modified vaccinia virus Ankara (MVA) is a VACV derivative that gained extensive attenuation by routine passaging in cell culture. MVA lost many immunomodulatory genes including the poxin-encoding *B2R* and became a highly immunogenic virus and an attractive vaccine candidate. In agreement, MVA induction of IFN and other cytokines depends on STING [16,17], and STING is crucial in the establishment of efficient cytotoxic T cell responses during MVA immunisation [18]. Given the remarkable contribution to virulence by vSlfn, poxin inactivation is likely to contribute to MVA attenuation. Surprisingly, poxin inactivation also occurs in VARV, the causative agent of the devastating smallpox disease. A common pattern of gene inactivation and gene loss is observed in OPXV with narrow host range, possibly as a result of host adaptation, and recent evidence comparing approximately 1,000-year-old specimens with modern-day VARV support a similar trend [19]. There is no known animal reservoir for VARV, so inactivation of the Slfn domain could be explained by its adaptation to human-to-human transmission. The inactivation of the poxin domain is, however, more puzzling and suggests that VARV evolved to avoid DNA sensing activation or to block it by alternative methods.

### Absence of poxin

Given the cytosolic nature of poxviruses, the absence of poxin in multiple poxvirus species raises questions about how these species contend with host DNA sensing pathways. Multiple bird poxviruses exist and are grouped in the avipoxvirus genus (Fig 1). Although the avian immune system differs from the mammalian counterpart, recent work in chicken cells has demonstrated that the cGAS/STING pathway is active against avian poxvirus infection [20]. This suggests that a selective pressure to evade cGAS/STING activation may exist in avian poxviruses. However, although many birds prey on insects like bats, no horizontal transmission of poxin genes from insect to bird poxviruses has occurred. The absence of poxin is also noteworthy in many mammalian poxviruses including parapoxviruses, leporipoxviruses, or capripoxviruses (Fig 1). Some of these, like the leporipoxvirus myxoma virus (MYXV), can be highly virulent and have undergone genome recombination and gene transfer events in the wild [21]. Further studies are therefore required to address how, in the absence of poxin, these viral species suppress DNA sensing and, if occurring, how this contributes to immune evasion and virulence. The absence of cGAMP nucleases is also significant in mammalian hosts. While insects encode several poxin genes, some of which are extracellular, there is no evidence for mammalian strategies to increase cGAMP turn-over beyond the plasma membrane enzyme ENPP1 [22]. Is, then, cGAMP particularly toxic in insects? Do mammals instead regulate cGAMP-induced effector mechanisms, such as IFN, by-passing the need for cGAMP regulation? Is cGAS activation in mammals much more tightly controlled than insect cGAMP-producing enzymes? Certainly, there is growing evidence for complex regulation of human and mouse cGAS [23], and the identification of the elusive cGAS-like enzymes in insects will enlighten both insect and mammalian immune sensing. Many inflammatory disorders are directly and indirectly associated with aberrant cGAS and DNA sensing responses that result in cGAMP production and STING activation [24]. Studies on viral immunosuppressive mechanisms have the potential to uncover novel therapeutic strategies to modulate pathophysiology in disease, besides revealing the potency of unleashed DNA sensing responses during infection.
